# ECM Modifications Driven by Age and Metabolic Stress Directly Promote Vascular Smooth Muscle Cell Osteogenic Processes

**DOI:** 10.1161/ATVBAHA.124.321467

**Published:** 2025-01-16

**Authors:** Meredith Whitehead, Maria Faleeva, Rafael Oexner, Susan Cox, Lukas Schmidt, Manuel Mayr, Catherine M. Shanahan

**Affiliations:** 1 British Heart Foundation Centre of Research Excellence, School of Cardiovascular and Metabolic Medicine & Sciences (M.W., M.F., R.O., L.S., M.M., C.M.S.), King’s College London, United Kingdom.; 2 Randall Centre for Cell & Molecular Biophysics, Faculty of Life Sciences & Medicine (S.C.), King’s College London, United Kingdom.

**Keywords:** collagen, DNA damage, gene expression, hydroxyapatites, phenotype

## Abstract

**BACKGROUND:**

The ECM (extracellular matrix) provides the microenvironmental niche sensed by resident vascular smooth muscle cells (VSMCs). Aging and disease are associated with dramatic changes in ECM composition and properties; however, their impact on VSMC phenotype remains poorly studied.

**METHODS:**

Here, we describe a novel in vitro model system that utilizes endogenous ECM to study how modifications associated with age and metabolic disease impact VSMC phenotype. ECM was synthesized using primary human VSMCs and modified during culture or after decellularization. Integrity, stiffness, and composition of the ECM was measured using superresolution microscopy, atomic force microscopy, and proteomics, respectively. VSMCs reseeded onto the modified ECM were analyzed for viability and osteogenic differentiation.

**RESULTS:**

ECMs produced in response to mineral stress showed extracellular vesicle–mediated hydroxyapatite deposition and sequential changes in collagen composition and ECM properties. VSMCs seeded onto the calcified ECM exhibited increased extracellular vesicle release and Runx2 (Runt-related transcription factor 2)-mediated osteogenic gene expression due to the uptake of hydroxyapatite, which led to increased reactive oxygen species and the induction of DNA damage signaling. VSMCs seeded onto the nonmineralized, senescent ECM also exhibited increased Runx2-mediated osteogenic gene expression and accelerated calcification. In contrast, glycated ECM specifically induced increased ALP (alkaline phosphatase) activity, and this was dependent on RAGE (receptor for advanced glycation end products) signaling with both ALP and RAGE receptor inhibition attenuating calcification.

**CONCLUSIONS:**

ECM modifications associated with aging and metabolic disease can directly induce osteogenic differentiation of VSMCs via distinct mechanisms and without the need for additional stimuli. This highlights the importance of the ECM microenvironment as a key driver of phenotypic modulation acting to accelerate age-associated vascular pathologies and provides a novel model system to study the mechanisms of calcification.

**Highlights**

- The initial deposition of hydroxyapatite in the ECM (extracellular matrix) is mediated by extracellular vesicles.
- Calcification of the ECM induces osteogenic differentiation of healthy vascular smooth muscle cells by mineral uptake–induced oxidative stress and activation of the DNA damage repair pathway.
- Age-associated changes to the ECM enhance vascular calcification by directly promoting osteogenic differentiation of vascular smooth muscle cells.
- Accumulation of advanced glycation end products in the ECM accelerates calcification by increasing alkaline phosphatase expression and activity via receptor for advanced glycation end products.

Aging is the strongest risk factor for cardiovascular morbidity and mortality.^1^ Vascular aging manifests as a multifaceted process marked by gradual changes to the structure and rigidity of the vessel wall and the emergence of detrimental pathologies such as calcification, atherosclerosis, amyloidosis, and aneurysms. It is associated with both cellular senescence and modifications to the composition and physical properties of the ECM (extracellular matrix), which impact vessel biomechanics and the function of resident cells.^2^ This results in defective ECM-cell signaling, which may act in a feed-forward cycle to drive further pathological change.^3^

Typical features of the aging ECM include matrix breakdown, cross-linking, and glycation, as well as changes in the composition and ratios of collagens, elastin, proteoglycans, and their associated binding proteins.^3,4^ These ECM modifications are driven by both physical and chemical damage, as well as by cell-mediated processes, and lead to the development of age-associated pathologies, such as vascular calcification.^5,6^ Metabolic disturbances accelerate changes in the ECM that can mimic features of chronological aging. In chronic kidney disease (CKD), dysregulated mineral metabolism drives accelerated vascular smooth muscle cell (VSMC) death and senescence and accumulation of calcium (Ca) in the vessel wall.^7^ In diabetes, advanced glycation end products (AGEs), formed through nonenzymatic protein glycosylation due to increased levels of circulating glucose, accumulate on collagen and elastin.^8^ Correspondingly, patients with diabetes and CKD show accelerated calcification.^9,10^

Vascular calcification is characterized by the deposition of calcium (Ca) and phosphate (P) salts in the ECM, which ultimately can take the form of hydroxyapatite (HA). The ECM can calcify via physicochemical or active processes mediated by VSMCs, with these mechanisms not mutually exclusive.^11–13^ In its earliest form, calcification is commonly observed as round, punctate crystal aggregates, often in association with extracellular vesicle (EV) deposition.^14,15^ Calcifying VSMCs contribute to mineralization by secreting EVs and expressing bone-associated, osteogenic proteins including Runx2 (Runt-related transcription factor 2),^16^ ALP (alkaline phosphatase), and BMP2 (bone morphogenetic protein 2) that play roles in regulating the mineralization process.^17^ Minerals can also be directly deposited onto ECM components, such as collagen and elastin, which act as scaffolds for both EV and Ca binding.^13,18^

Current in vitro models of VSMC calcification rely by necessity on agents being added to cell cultures in solution. This includes the induction of mineralization by exposure of VSMCs to supraphysiological levels of Ca and P or their crystals, including calciprotein particles, to model mineral disturbances in CKD. Alternatively, mineralization can be induced by treatment with β-glycerophosphate, which requires breakdown by ALP to release P, thus selecting for VSMCs with this osteogenic activity. In these contexts, additional factors are also added, including soluble peptides derived from ECM breakdown or AGEs, to mimic matrix modifications.^19–21^ While these studies have provided important insights into promoters and mechanisms of calcification, they do not fully recapitulate the in vivo situation where VSMCs encounter a complex modified ECM with or without the embedded mineral.

Clinical studies have shown that, once calcification can be detected, it progresses rapidly, leading to the suggestion that calcification exacerbates calcification; however, the mechanisms driving this apparent acceleration remain unclear.^5,6^ Some studies have suggested that the accumulation of HA in the ECM is required to induce osteogenic differentiation of VSMCs, with this feed-forward cycle responsible for accelerated mineralization. However, in human disease, the relationships between HA accumulation, osteogenic differentiation, and the contributions of ECM modifications to these processes have been difficult to dissect. Therefore, with these controversies and limitations in mind, we set out to establish a more physiologically relevant in vitro model whereby endogenously produced ECM could be used to study how VSMC-ECM interactions might contribute to calcification. Our results demonstrate that specific age and metabolically induced ECM modifications have a profound effect on VSMC phenotype and can directly induce osteogenic differentiation of healthy VSMCs. This occurs via multiple pathways and in the presence or absence of embedded mineral, highlighting a key role for the ECM microenvironment in accelerating calcification and the heterogeneity of calcification pathways. This heterogeneity may account for the failure to develop therapeutics that block calcification and will prove informative for the future development of targeted treatments for this detrimental pathology.

## Materials and Methods

### Data

All supporting data are available within the article and its Supplemental Material.

### Cell Culture and Treatments

Primary VSMCs were isolated from explants of human aortic tissues from 5 young transplant donors; 35-year-old woman (04-35F-11A), 20-year-old man (05-20M-8A), 22-year-old man (05-22M-18A), 38-year-old woman (03-38F-9A), and 33-year-old woman (05-33F-5A). Visual examination and histological analysis showed that the vessels were healthy with no evidence of atherosclerosis. All experiments were performed using at least 3 biological replicates unless stated. Human materials were handled in compliance with the Human Tissue Act (2004; United Kingdom) and with ethical approval from the Research Ethics Committee (reference: 13/LO/1950). Cells were cultured in M199 medium supplemented with 20% fetal bovine serum and 1% penicillin-streptomycin-glutamine at 37 °C with 5% CO_2_.

### o-Cresolphthalein Assay

The deposition of Ca was measured using an o-cresolphthalein assay, as previously described.^22^ After a 7- or 18-day Ca and P treatment, the cells were lysed and the remaining ECM was washed with Hanks’ balanced salt solution and the Ca was dissolved in 0.1 mol/L hydrochloric acid overnight at 4 °C. Ca concentration was quantified by adding o-cresolphthalein reagent in ammonium buffer. Absorption was measured using a Tecan spectrophotometer microplate reader at 540 nm and quantified using a standard curve of calcium chloride. The Ca concentration was normalized to protein concentration, measured with a detergent compatible protein assay, and calculated relative to the control condition.

### ECM Synthesis

For synthesis of ECM, plates or glass coverslips were coated with gelatin, cross-linked with glutaraldehyde, which was then quenched with ethanolamine, as described previously.^23^ Early passage (<12) VSMCs were seeded at high confluency and cultured in complete media supplemented with 50 μg/mL sodium L-ascorbate. To induce ECM mineralization, 2.7 mmol/L Ca and 2.5 mmol/L P was added during ECM synthesis, for 7 days (early calcification) or 18 days (late calcification). To mimic the aging ECM, replicative senescent VSMCs at late passages (passages 22–24) were seeded for ECM synthesis and compared with early passage, proliferative VSMCs (passages 10–12), a model previously described and validated.^24,25^ To remove the VSMCs and decellularize the ECM, extraction buffer (0.1% Triton X-100, 20 mmol/L NH_4_OH in PBS) was added for 5 minutes at 37 °C before washing 3× in PBS to remove cell debris. Removal of cells was verified using DAPI (4′,6-diamidino-2-phenylindole) staining. The decellularized ECM was glycated by incubating with 0.66 mol/L D-ribose in Hanks’ balanced salt solution for 14 days, based on a previously published protocol.^26^ The control ECM was treated with Hanks’ balanced salt solution alone for 14 days. Early passage VSMCs were then seeded on control, calcified, senescent, or glycated ECM to investigate the effect of ECM on VSMC phenotype and function. To inhibit RAGE (receptor for AGEs) on the glycated ECM, cells were treated with 1 μmol/L FPS ZM1 (Merck; 553030), while ALP was inhibited with 100 μmol/L levamisole (Merck; 31742)

### Atomic Force Microscopy

Atomic force microscopy (AFM) was performed using a tetrahedral shaped cantilever with a silicone tip with a spring constant of 0.081 N/m, tip height of 4 to 5 µm (HYDRA-6v-200WG; Applied Nanostructures, CA), as previously described.^25^ Stiffness of the ECM was recorded by contact mode, measuring force spectroscopy in liquid. The set point was 5 nN with 14 µm Z length and 10 µm/s extend speed. Data were acquired and analyzed using the JPK Nanowizard 3 software.

### Immunofluorescence and Quantification

Decellularized ECM with or without VSMCs seeded was fixed in 4% paraformaldehyde in PBS for 10 minutes followed by washing and blocking in 3% BSA in PBS for 1 hour. Primary antibodies were added for 1 hour before washing in PBS: CD63 (Santa Cruz; sc-15363, 1:500), fibronectin (Abcam; ab2413, 1:1000), phosphorylation of histone H2AX on serine 139 (γH2AX; Cell Signaling Technology; 2577, 1:200), RAGE (Abcam; ab37647, 1:200), and carboxymethyl lysine (CML; Abcam; ab125145, 1:200). Alexa Fluor fluorescent dye–conjugated secondary antibodies (Invitrogen) were added for 1 hour: 546 goat anti-rabbit (A11035, 1:400), 488 goat anti-rabbit (A11008, 1:400), and 546 donkey anti-mouse (A11036, 1:400). OsteoImage reagent (Lonza; PA-1503) was used to stain HA according to the manufacturer’s instructions. Briefly, OsteoImage was diluted 1:100 in staining buffer, added to the coverslips, and incubated for 30 minutes. Phalloidin (Invitrogen; A30107, 1:400) was used to stain F-actin. Phalloidin-647 was diluted in PBS and incubated with the coverslips for 30 minutes before washing. DAPI (Sigma; D9542, 1:10 000) was used to stain nuclei for 2 minutes, and following washing, the coverslips were mounted using Mowiol mounting medium. Z stacks of the ECM were imaged using the Nikon A1R confocal microscope with NIS-Elements software. For superresolution microscopy, the ECM was imaged using a Nikon iSIM with NIS-Elements software. The area of extracellular HA and CD63 deposits or accumulation of DNA damage foci was quantified using an automatic threshold method in Fiji.

### CD63 Beads Assay and FACS

Quantification of CD63- and CD81-positive EV secretion was performed by a CD63 beads assay and FACS analysis, adapted from a previously described protocol.^27^ A concentration of 16.5 μmol/L 3-OMS (3-o-methylsphingomyelin; Enzo; BML-SL225-0005) was used as an inhibitor of EV secretion. Anti-human CD63 antibody (BD Biosciences; 556019) was immobilized on 4 µm aldehyde-sulfate beads (Invitrogen; A37304) and incubated with conditioned media overnight at 4 °C. The beads were washed with 2% BSA twice and incubated with phycoerythrin-conjugated CD81 antibody (BD Pharmingen; 555676, 1:50) for 1 hour. Following washing, the beads were resuspended in PBS and analyzed using a BD Accuri C6 flow cytometer. Arbitrary units were calculated using FlowJo as mean fluorescence units multiplied by the percentage of positive beads. Values were normalized to cell number, counted using NC3000 NucleoCounter.

### RNA Isolation and Reverse Transcription-Quantitative PCR

RNA from VSMCs seeded on plastic, control ECM or calcified ECM was collected using STAT60 and isolated using a phenol-chloroform extraction method. cDNA was synthesized using Mu-MLV reverse transcriptase with random and oligo primers, RNAse inhibitor and dNTPs (all from Promega). qPCR was performed in triplicates using qPCRBIO SyGreen Mix and run in the StepOnePlus Real Time PCR system using the following primers: *GAPDH* (QT00079247), *Runx2* (QT00020517), *α-SMA* (α-smooth muscle actin; forward [FW]: 5′-TGA CAA TGG CTC TGG GCT CTG TAA-3′; reverse [RV]: 5′-TCC ACG GTA GTG CCC ATC ATT CTT-3′), *SM22α* (smooth muscle protein 22α; FW: 5′-TTG AAG GCA AAG ACA TGG GAG CAG-3′; RV: 5′-TTC GTC ACC CAC GTA GCT GTC TTT-3′), *ALP* (FW: 5′-CACCAGCAAGAAGAAGCCTTTG-3′); RV: 5′-ACGAGCTGAACAGGAACAACGT-3′), *BMP2* (QT00012544), *BSP* (bone sialoprotein; QT00115304), *SMPD3* (sphingomyelin phosphodiesterase 3; FW: 5′-GCCTATCACTGTTACCCCAAC-3′; RV: 5′-GACGATTCTTTGGTCCTGAGG), and *RAGE* (FW: 5′-CACCTTCTCCTGTAGCTTCAGC-3′; RV: 5′-AGGAGCTACTGCTCCACCTTCT-3′). Expression of target genes was calculated using the 2^−ΔΔCt^ method, with *GAPDH* expression used for normalization.

### Western Blotting

ECM or cell lysates were homogenized in radioimmunoprecipitation assay buffer with protease inhibitor cocktail, sonicated, and centrifuged at 16 000*g* for 15 minutes at 4 °C. Loading buffer (40% glycerol, 240 mmol/L Tris-HCl pH 6.8, 8% SDS, 0.04% bromophenol blue, 50 mmol/L dithiothreitol) was added and the samples boiled at 95 °C for 5 minutes. The proteins were separated by SDS-PAGE using 4% to 15% precast polyacrylamide gels (Biorad; 4561086) and transferred to polyvinylidene fluoride membrane by semidry transfer. The membrane was blocked with 5% skimmed milk in PBS-Tween (0.05%) for 1 hour at RT. Primary antibodies were diluted in milk and incubated with the membrane overnight at 4 °C: fibronectin (Abcam; ab2413, 1:500), RAGE (Abcam; ab37647, 1:500), and CML (Abcam; ab125145, 1:500). Following washing in PBS-Tween, the membranes were incubated with fluorescently conjugated secondary antibodies for 1 hour at RT: IRDye 800CW donkey anti-rabbit (LI-COR; 926-32213, 1:10 000) and IRDye 680RD donkey anti-mouse (LI-COR; 926-68072, 1:10 000). The membranes were imaged using Odyssey (LI-COR) and quantified through Image Studio. The membranes were stripped in a low-pH glycine buffer (1.5% glycine, 0.5% SDS, 1% Tween-20 pH 2.2) for 20 minutes, before washing and reblocking.

### Proteomics and Bioinformatics Analysis

Proteomics was performed on decellularized control, early or late calcified ECM from VSMC isolates from a 35-year-old woman, 22-year-old man, and 20-year-old man. The ECM was precipitated and deglycosylated with enzymes (chondroitinase, heparinase, keratinase, PNGase F, O-deglycosidase, and debranching enzymes) before denaturation using 6 mol/L urea/2 mol/L thiourea, reduction with 10 mmol/L dithiothreitol, and alkylation with 50 mmol/L iodoacetamide. Following acetone precipitation and drying, proteins were resuspended in 100 mmol/L triethylammonium bicarbonate and digested with 0.8 μg trypsin/Lys-C overnight at 37 °C. Peptides were cleaned using C18 resin columns and resuspended in 2% acetonitrile, 0.05% trifluoroacetic acid in water before injection into a nanoflow liquid chromatography system (Dionex UltiMate 3000 RSLCnano; Thermo Scientific). The eluate was then sprayed into a Q Exactive HF mass spectrometer (Thermo Scientific) and analyzed by data-dependent acquisition–mass spectrometry.

Proteome Discoverer software (version 2.3.0.523) was used to search raw data files against human and bovine UniProtKB/Swiss-Prot databases using Mascot. To account for differences in protein amounts during sample preparation, abundance values were normalized to the total peptide amount of the most abundant sample. Visualization of the data was performed using the gplots package in R Studio and GraphPad Prism. Protein-protein interaction networks and functional enrichment tests were done in Cytoscape 3.10.1 with attached STRING application (app). Proteomics data are available on an Open Science Framework repository at doi: 10.17605/OSF.IO/5VY4S.

### Cell Vitality Assay

Cell vitality was assessed by quantification of free, reduced thiols. VSMCs were lysed from plastic, control, or calcified ECM and centrifuged at 1000*g* for 5 minutes. The cell pellet was resuspended in Solution 5 (ChemoMetec; 910-3005) diluted 1:20 in media. Solution 5 contains VitaBright-48, which fluoresces when bound to reduced thiols, acridine orange to stain all cells, and propidium iodide to stain dead cells. The cell suspension was added to slides and analyzed using a NucleoCounter NC3000. The results were analyzed by applying a gating strategy to VSMCs cultured on plastic to identify clear populations of healthy, unhealthy, and dead VSMCs. This gating was then applied to all other samples.

### Oxidative Stress Analysis

Accumulation of reactive oxygen species (ROS) was measured using a Cellular ROS Assay Kit (Abcam; ab186027) according to the manufacturer’s instructions. N-acetylcysteine (NAC; Merck; A9165) was used at 10 μmol/L to decrease oxidative stress. VSMCs were seeded on plastic, control ECM, or calcified ECM; the ROS Red Working Solution was added; and the cells incubated for 1 hour at 37 °C. The fluorescence intensity was monitored (excitation/emission, 520/605 nm) using a Tecan spectrophotometer.

### Calcein Staining and Quantification of Uptake

The control and calcified matrices were stained with calcein (Sigma; C0875), a Ca-dependent fluorescent molecule. To visualize Ca uptake from the ECM, the matrices were incubated with 0.2% calcein solution (1% NaHCO_3_ in 0.9% normal sterile saline solution) for 20 minutes at RT. Following washing with PBS, VSMCs were seeded for immunofluorescent (IF) staining and treated with 0.1 μmol/L wortmannin (Merck; W1628) to inhibit endocytosis, as previously described.^28^ To quantify the uptake, VSMCs were trypsinized, pelleted, and incubated with EDTA for 5 minutes to remove surface-bound HA. The cells were then lysed by sonication to measure intracellular calcein. The fluorescence intensity was measured using a Tecan spectrophotometer. Calcein-labeled HA was used as a positive control.

### Statistical Analysis

The results are presented as mean±SD. Normality for all data was tested using the Shapiro-Wilk test before comparison analysis. An unpaired Student *t* test was used to compare 2 independent groups with >10 samples or a Mann-Whitney *U* test for groups with <10 samples or groups that did not pass the normality test. For comparison of multiple groups with 1 independent factor, a Kruskal-Wallis test was performed. Where normality was validated in sample sizes >10, 1-way ANOVA with Tukey post hoc test was used. A 2-way ANOVA with Tukey post hoc test was used to analyze data with 2 independent factors. The equal variance assumption was satisfied before using parametric tests. The results were described as statistically significant when *P*<0.05. Data were obtained from a minimum of 3 independent experiments with a minimum of 3 technical replicates.

## Results

### Calcification of the ECM Is Mediated by EVs and Drives ECM Stiffening

**Figure 1.**
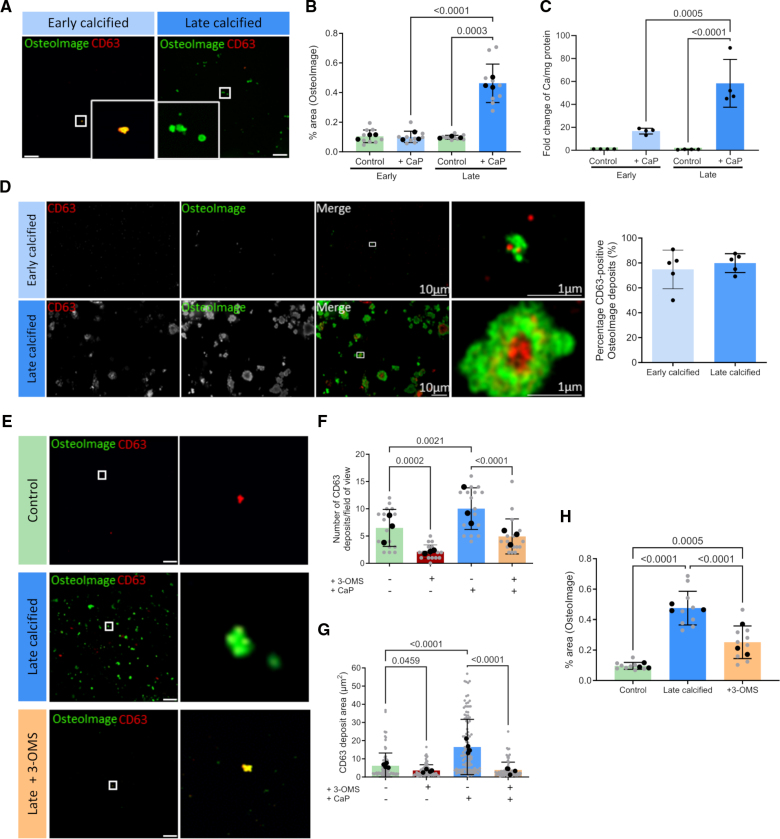
**ECM (extracellular matrix) mineralization is mediated by small extracellular vesicles (EVs). A**, Immunofluorescent (IF) staining of early and late calcified ECM showing accumulation of hydroxyapatite (HA), detected by OsteoImage reagent, and CD63-positive EVs. Scale bar is 20 μm. **B**, Quantification of percentage area of OsteoImage staining in control, early, and late calcified ECM using the threshold method (n=9). **C**, Calcium content of the ECM was measured by o-cresolphthalein assay and showed 20-fold increase in calcium in the early calcified ECM and 60-fold increase in the late calcified ECM, compared with control counterparts (n=4). **D**, Superresolution microscopy showed OsteoImage colocalized with CD63-positive EVs at the core of the deposits, and quantification indicated 75% to 80% of OsteoImage deposits colocalized with CD63 in both early and late calcified ECM (n=5). **E**, EV secretion was decreased with 3-OMS (3-o-methylsphingomyelin) during ECM synthesis, and IF showed a decrease in OsteoImage staining in the late calcified ECM with 3-OMS treatment compared with the untreated late calcified ECM. Scale bar is 20 μm. Quantification of CD63 staining confirmed a decrease in the (**F**) number of EV deposits and (**G**) size of EV deposits in the ECM with 3-OMS treatment (n=3 with minimum 5 fields of view). **H**, Accumulation of HA in the late calcified ECM was reduced with 3-OMS treatment, confirmed by quantification of OsteoImage staining (n=9). All data are presented as mean±SD. All experiments were performed in the 04-35F-11A donor.

To model ECM modifications induced under conditions of dysregulated mineral metabolism, VSMCs were primed to produce ECM in the presence or absence of elevated levels of Ca and P for 7 or 18 days. Imaging of the decellularized ECM under bright-field microscopy detected no mineral deposition in the control or 7-day ECM while extensive mineralization was present at 18 days (Figure S1A). IF staining and quantification with OsteoImage detected occasional small puncta of HA at 7 days, while at 18 days, there was extensive deposition of circular-shaped crystals averaging ≈5 μm^2^ in area (Figure 1A and 1B). Despite the low levels of HA detectable in the ECM at 7 days, o-cresolphthalein assay showed a significant 20-fold increase in Ca, compared with a 60-fold increase in the 18-day ECM (Figure 1C). Thus 7-day ECM was designated early calcification and 18-day ECM as late calcification.

To study the origin of the calcified deposits, superresolution microscopy was performed. At 7 days, staining with the tetraspanin CD63 showed small, round EVs averaging 100 nm in diameter colocalizing with the sparse HA deposits, which appeared to surround the EVs. At 18 days, the more extensive HA deposits surrounded a core of larger CD63-positive and TSG101 (tumor susceptibility gene 101)-positive vesicle aggregates (Figure 1D; Figure S1B). At both time points, 75% to 80% of OsteoImage deposits colocalized with CD63, indicating the majority of the HA in the ECM is formed around EVs rather than directly onto ECM components (Figure 1D). Consistent with this idea, we observed that Ca and P treatment rapidly increased EV secretion from VSMCs at 24 hours, and this preceded osteogenic differentiation, which was only evident at the late calcification time point (Figure S1C and S1D). Treatment with 3-OMS blocked EV release and decreased the number and size of CD63-positive areas in the ECM under control and calcifying conditions (Figure 1E through 1G; Figure S1D and S1E). OsteoImage quantification showed this reduction in EV deposition corresponded to significantly reduced accumulation of HA in the late calcified ECM (Figure 1H).

### Calcifying Conditions Induced Changes in ECM Properties and Composition

**Figure 2.**
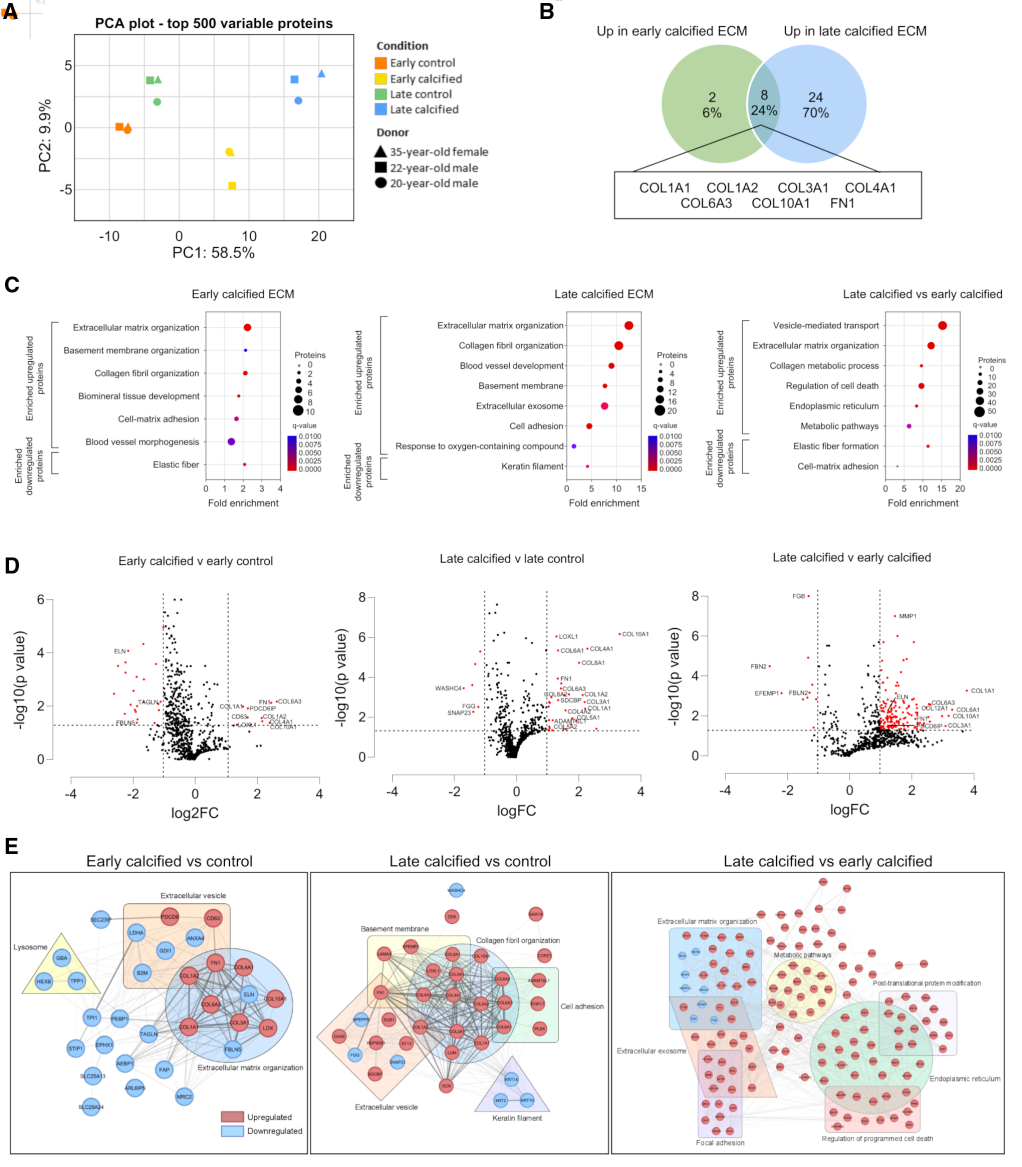
**Calcifying conditions drive changes in ECM (extracellular matrix) composition. A**, Principal component analysis (PCA) of ECM proteomics, identifying distinct clusters with early and late calcified samples forming separate clusters to the control matrices that clustered together (n=3). **B**, Venn diagram showing proteins upregulated in both early and late calcified ECM compared with control counterparts. **C**, Gene ontology (GO) identified several significantly altered biological pathways in the early calcified vs control, late calcified vs control, and late calcified vs early calcified. **D**, Volcano plots of differentially expressed proteins in early calcified vs control, late calcified vs control, and late calcified vs early calcified. Cutoffs were set at log fold change (FC) <−1 to >1 and −log_10_(*P*)>1.3. **E**, Protein-protein interaction networks for early calcified vs control, late calcified vs control, and late calcified vs early calcified. Data are presented from 04-35F-11A, 05-20M-8A, and 05-22M-18A donors.

To determine the changes in ECM composition induced by mineral stress, proteomics was performed on decellularized early and late calcified ECM and control counterparts. Principal component analysis showed the calcified matrices formed separate clusters compared with the early and late controls, which clustered together (Figure 2A). This clustering was reiterated when differentially expressed proteins were visualized using a heatmap (Figure S2A). Analysis of matrisomal proteins indicated that in both early and late calcified matrices, there were similar increases in the proportion of collagens, proteoglycans, and other ECM modifiers within the matrix (Figure S2B). Upregulation of collagens was observed in both early and late calcified ECM compared with their controls, with 8 proteins significantly upregulated at both time points including collagens 1, 3, 4, 6, and 10, fibronectin, and LOXL1 (lysyl oxidase like 1), all of which have been implicated in calcification or bone and cartilage formation (Figure 2B).

Gene ontology analysis comparing the early calcified and control ECM identified several upregulated pathways, including ECM and basement membrane organization, biomineral tissue development (involving COL1A1 [collagen type 1-α1], COL1A2 [collagen type 1-α2], LOXL1, and ECM1 [ECM protein 1]) and cell-matrix adhesion (Figure 2C). Similar pathway changes were observed when comparing the late calcified ECM with the control, with the addition of extracellular exosome and response to oxygen containing compound. Interestingly, when comparing early and late calcified matrices, there was significant downregulation of proteins involved in elastic fiber formation (FBLN2 [fibulin 2], FBN2 [fibrillin 2], EFEMP1 [EGF-containing fibulin-like ECM protein 1], and MFAP5 [microfibrillar-associated protein 5]), while upregulated pathways included metabolic pathways, regulation of cell death, and endoplasmic reticulum, suggesting VSMC dysfunction increases at late stages of calcification.

Volcano plots showed that the majority of significant differences in proteins were observed in the late calcified compared with the early calcified ECM (Figure 2D), with COL1A1 being the most significantly upregulated protein. In all comparisons, there was also upregulation of EV markers, including CD63, PDCD6IP (programmed cell death 6-interacting protein), and SDCBP (syntenin-1), as well as modifiers of the ECM, such as LOXL1, MMP1 (matrix metalloproteinase 1), and ADAMTSL1 (a disintegrin and metalloproteinase with thrombospondin motifs-like protein 1).

Protein-protein interaction networks for significantly altered proteins identified collagen fibril organization, basement membrane, and EVs as important groups that interact and intersect (Figure 2E). In the late versus early calcified interaction network, proteins involved in ECM organization, exosomes, and focal adhesions were also connected, highlighting an important relationship between VSMCs, the ECM, and EVs.

### Calcified ECM Can Induce Osteogenic Processes in VSMCs

**Figure 3.**
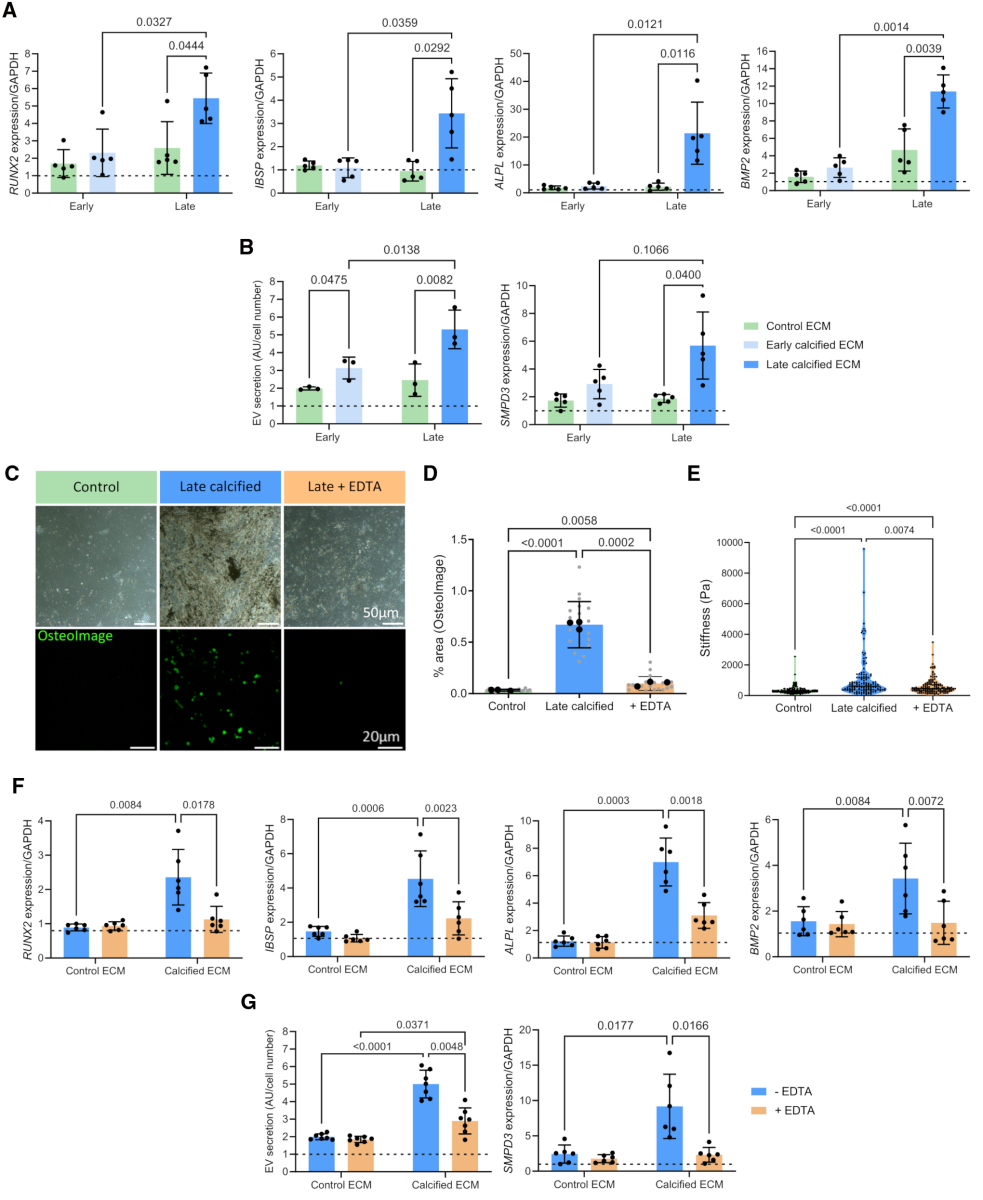
**ECM (extracellular matrix) calcification induces osteogenic differentiation of vascular smooth muscle cells. A**, Reverse transcription-quantitative PCR (RT-qPCR) of osteogenic markers *Runx2* (Runt-related transcription factor 2), *BSP* (bone sialoprotein), *ALP* (alkaline phosphatase), and *BMP2* (bone morphogenetic protein 2) in vascular smooth muscle cells (VSMCs) seeded on control, early, and late calcified ECMs. Expression was normalized to cells seeded on tissue culture plastic, indicated by dashed line (n=5). **B**, Quantification of CD63- and CD81-positive extracellular vesicles (EVs) from cells seeded on ECM relative to plastic control (n=3), and the corresponding expression of the EV secretion regulator, *SMPD3* (sphingomyelin phosphodiesterase 3), in cells on ECM (n=5). **C**, Hydroxyapatite (HA) was dissolved and removed from the late calcified ECM using EDTA and visualized by bright-field and immunofluorescence (IF) microscopy. **D**, Quantification of OsteoImage IF staining in control and late calcified ECM with and without EDTA treatment confirmed the late calcified ECM had been decalcified (n=3). **E**, Atomic force microscopy (AFM) measured the stiffness of the control (n=168), late calcified (n=168), and decalcified matrices (n=139). **F**, Expression of osteogenic markers Runx2, ALP, BSP, and BMP2 in VSMCs seeded on late calcified ECM and decalcified (+EDTA) ECM, normalized to plastic control (n=6). **G**, Quantification of EV secretion and expression of nSMase 2 was measured in cells seeded on control, late calcified ECM, and decalcified ECM, normalized to plastic control (n=6). All data are presented as mean±SD. All experiments were performed in the 04-35F-11A donor.

We next examined how VSMCs respond to the calcified ECM. Bright-field microscopy showed VSMCs could adhere to both calcified and control ECM with no indication that the calcified ECM was inducing cell death (Figure S3A and S3B). Expression of contractile markers, *α-SMA* and *SM22α*, was reduced on all the matrices due to the ECM being softer than plastic, as previously described (Figure S3C).^25^ Analysis of osteogenic gene expression showed that control ECM or early calcified ECM did not induce changes in expression of osteogenic markers. In contrast, there was marked upregulation of key osteogenic genes *Runx2,* *BSP*, *ALP*, and *BMP2* on the late calcified matrix (Figure 3A). This was accompanied by a marked increase in nuclear Runx2 protein shown by IF microscopy (Figure S3D).

Measurement of EV secretion in the conditioned media showed that VSMCs plated on control ECM increased EV secretion 2-fold compared with the plastic control (Figure 3B). EV secretion was further increased in VSMCs seeded on early calcified and late calcified ECM. This corresponded to increased expression on late calcified ECM of *SMPD3*, known to regulate EV secretion (Figure 3B).

Given the increased EV release observed on early calcified ECM compared with control ECM, we investigated whether calcification of VSMCs would be accelerated on this matrix in response to calcifying conditions. OsteoImage staining showed that calcifying conditions induced HA deposition on both matrices; however, there was a significant increase on the early calcified ECM compared with control (Figure S3E), suggesting ECM changes induced during the earliest phases of mineralization can create a procalcific environment.

To understand what component of the ECM was driving VSMC osteogenic change on the late calcified ECM, HA was removed with EDTA. IF staining and quantification showed a significant loss of HA from the ECM (Figure 3C and 3D). AFM showed that removal of HA also led to a significant decrease in stiffness of the ECM; however, the decalcified matrix remained stiffer than the control ECM, consistent with the upregulation of collagens and downregulation of elastic fibers quantified using proteomics. Seeding VSMCs onto the decalcified ECM significantly attenuated the increased expression of *Runx2*, *BSP*, *ALP*, and *BMP2* observed on the late calcified ECM (Figure 3F). There was also a significant decrease in EV secretion and *SMPD3* expression (Figure 3G). However, secretion of EVs on the decalcified ECM remained significantly higher than on control ECM, indicating an effect on EV secretion of the late calcified ECM independent of HA (Figure 3G).

### HA Uptake Induced VSMC Aging and Osteogenic Differentiation

**Figure 4.**
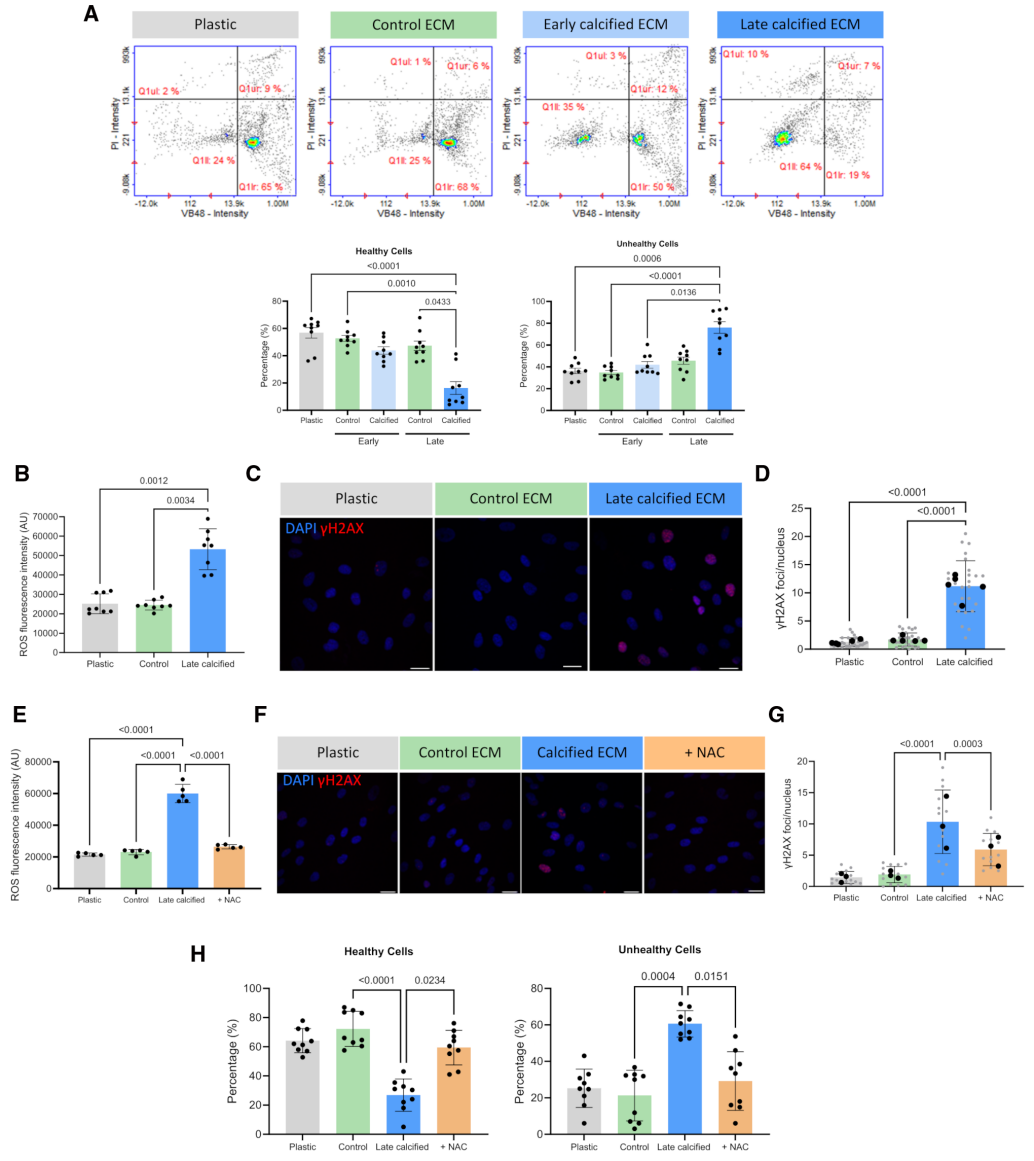
**ECM (extracellular matrix) calcification impacts cell vitality through the induction of DNA damage and oxidative stress. A**, Cell vitality was assessed in vascular smooth muscle cells (VSMCs) seeded on plastic, control ECM, and early and late calcified ECM by measuring reduced thiol levels and quantified as percentage healthy and unhealthy cells (n=9). **B**, Accumulation of reactive oxygen species (ROS) was observed in VSMCs seeded on late calcified ECM, compared with plastic and control ECM (n=8). **C**, Immunofluorescence (IF) of cells showing the accumulation of DNA damage, indicated by phosphorylation of histone H2AX on serine 139 (γH2AX) foci in the nucleus. Scale bar is 20 μm. **D**, Quantification of IF staining as number of γH2AX per nucleus (n=4). **E**, ROS generation was measured in VSMCs on late calcified ECM with antioxidant treatment using n-acetylcysteine (NAC) to confirm a reduction in oxidative stress (n=5). IF staining (**F**) and quantification of γH2AX foci (**G**) in VSMCs on late calcified ECM with NAC treatment (n=3). **H**, Quantification showing vitality of VSMCs seeded on plastic, control ECM, and late calcified ECM with or without NAC treatment (n=9). All data are presented as mean±SD. All experiments were performed in the 04-35F-11A donor.

To understand the mechanisms of increased osteogenic differentiation in response to the calcified ECM, we measured cell vitality by quantification of reduced cellular thiol levels. This showed a significant decrease in the percentage of healthy VSMCs and an increase in unhealthy VSMCs when seeded on late calcified ECM, compared with control or early calcified ECM, where no changes in cell vitality were observed (Figure 4A). The reduction in cell vitality corresponded to increased levels of ROS, measured by fluorescence intensity, in VSMCs seeded on the late calcified ECM with no effect on plastic or the control ECM (Figure 4B). The accumulation of DNA damage is a downstream effect of oxidative stress, and IF showed VSMCs seeded on late calcified ECM had extensive DNA damage (Figure 4C), with a 10-fold increase in the number of γH2AX foci per nucleus compared with the control or early calcified ECM that did not exhibit DNA damage (Figure 4D). Treatment of VSMCs on the calcified ECM with the antioxidant NAC reduced ROS production (Figure 4E) and led to a significant decrease in the number of γH2AX foci per cell (Figure 4F and 4G). This was associated with a decrease in the percentage of unhealthy and a corresponding increase in healthy VSMCs (Figure 4H).

**Figure 5.**
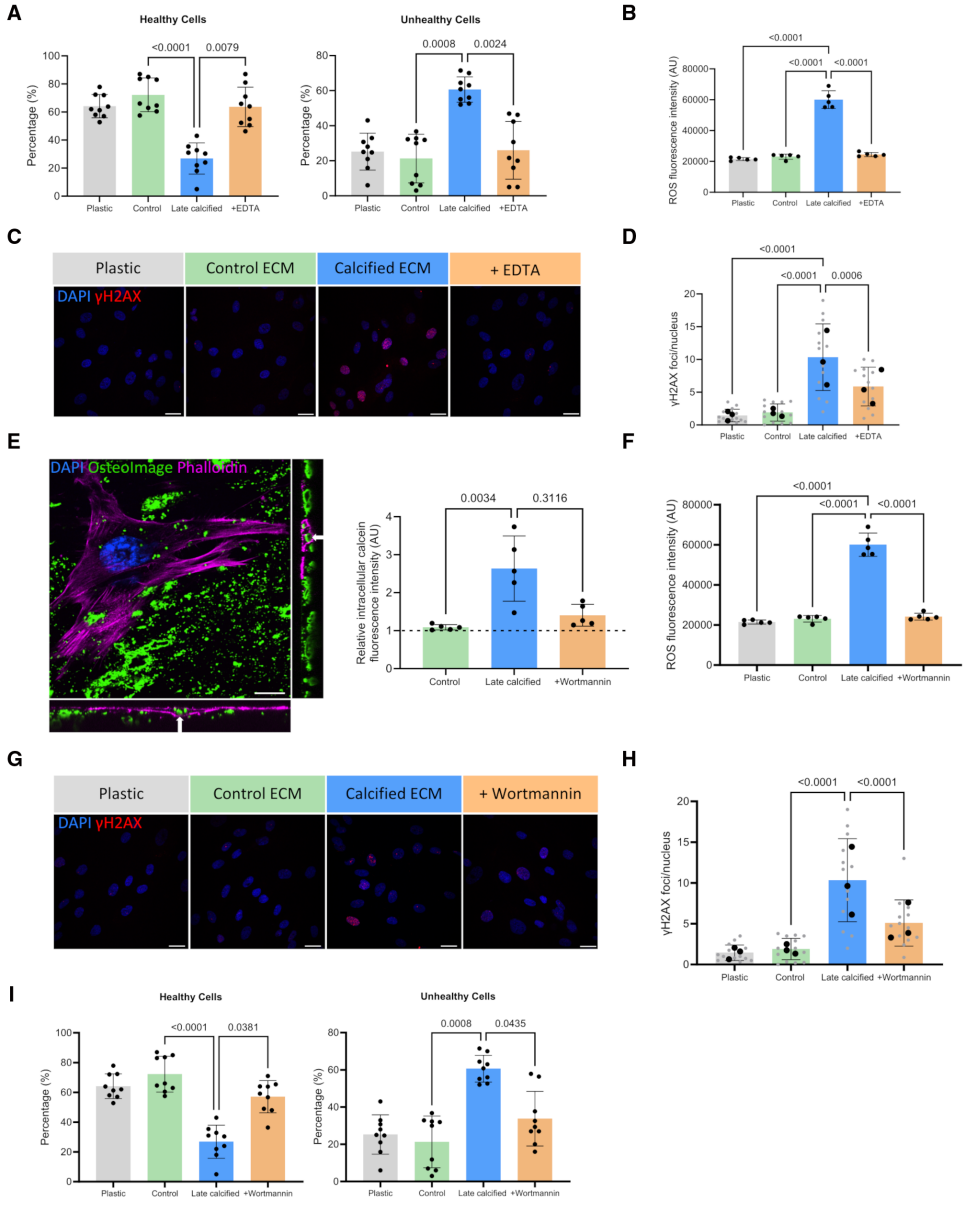
**Hydroxyapatite uptake from the ECM (extracellular matrix) drives changes in vascular smooth muscle cells (VSMCs). A**, Quantification of percentage healthy and unhealthy VSMCs seeded on plastic, control ECM, late calcified ECM, and decalcified ECM (+EDTA) measured by quantification of reduced thiol levels (n=9). **B**, Accumulation of reactive oxygen species (ROS) was measured by fluorescence intensity in VSMCs seeded on control ECM, late calcified ECM, and decalcified ECM (n=5). Immunofluorescent (IF) staining (**C**) and quantification of DNA damage (**D**), indicated by phosphorylation of histone H2AX on serine 139 (γH2AX) foci in the nucleus. Scale bar is 20 µm. **E**, IF of VSMCs seeded on late calcified ECM prestained with calcein, showing uptake of hydroxyapatite (HA) by the presence of intracellular calcein, indicated by white arrows. Intracellular calcein was quantified by trypsinizing and lysing VSMCs and measuring fluorescence intensity. Wortmannin was added as an endocytosis inhibitor and confirmed reduced uptake of calcein (n=5). Scale bar is 5 µm. **F**, Quantification of ROS in VSMCs seeded on plastic, control ECM, and late calcified ECM with or without wortmannin treatment (n=5). IF staining (**G**) and quantification of γH2AX foci (**H**) in VSMCs seeded on the ECM with wortmannin treatment (n=3). Scale bar is 20 µm. **I**, Quantification of the effect of wortmannin on vitality of VSMCs seeded on late calcified ECM (n=9). All data are presented as mean±SD. All experiments were performed in the 04-35F-11A donor.

To determine whether the HA was the cause of increased ROS production, VSMCs were again seeded on decalcified ECM. Removal of HA increased cell vitality and significantly reduced ROS production (Figure 5A and 5B) with a corresponding decrease in γH2AX foci (Figure 5C and 5D). To determine whether HA uptake from the ECM was driving ROS production, IF was performed on VSMCs seeded on mineralized matrices that had been prestained with the HA dye calcein. Intracellular calcein was detected by confocal microscopy in VSMCs, and treatment with the endocytosis inhibitor wortmannin decreased intracellular calcein (Figure 5E).^29^ This was associated with an increase in the percentage of healthy VSMCs and a reduction in the accumulation of ROS and γH2AX foci, suggesting a role for HA uptake in driving osteogenic change (Figure 5E through 5I).

### HA-Driven DNA Damage Signaling Induced VSMC Osteogenic Differentiation

**Figure 6.**
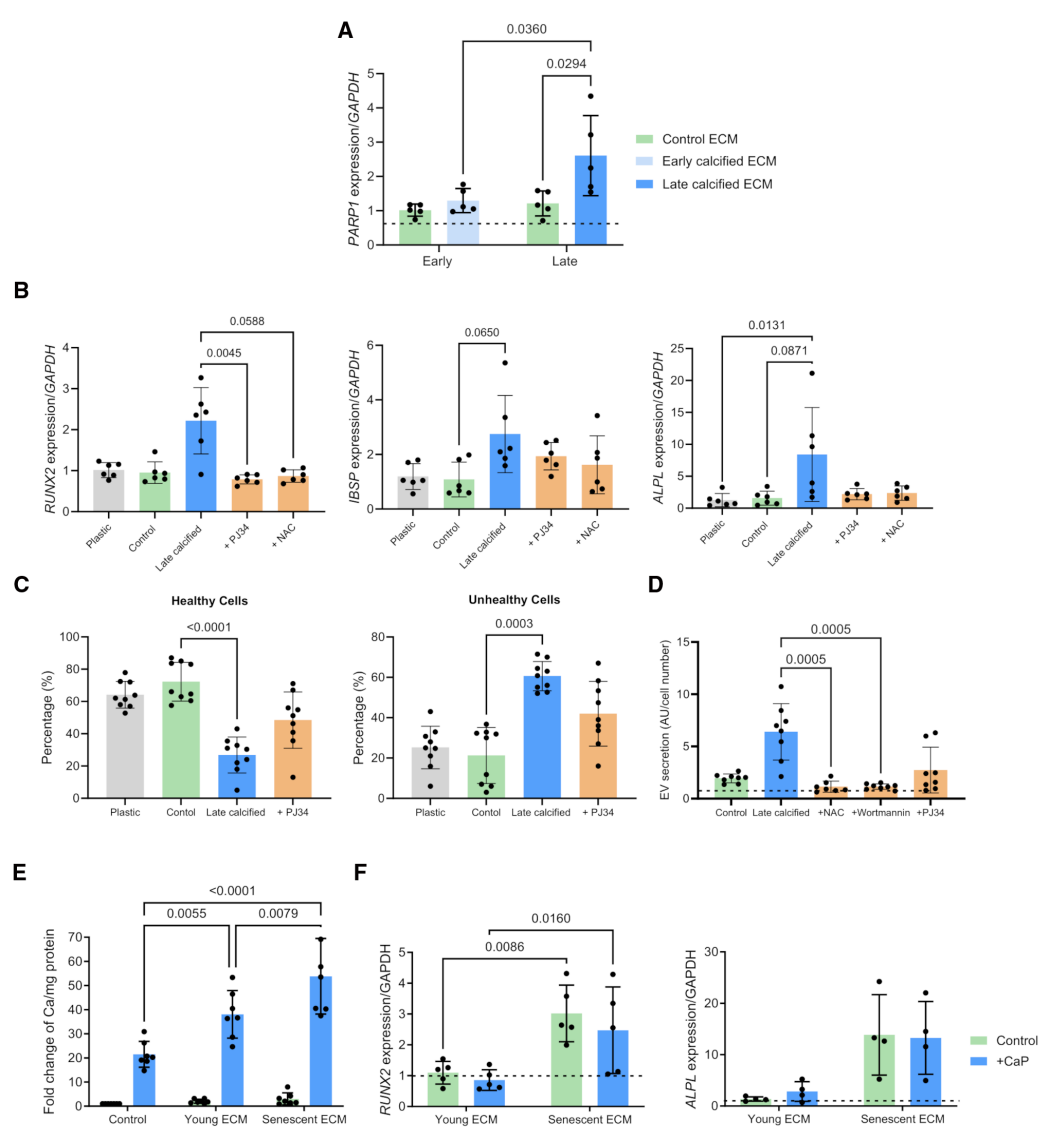
**Activation of the DNA damage repair pathway induces osteogenic differentiation. A**, Reverse transcription-quantitative PCR (RT-qPCR) analysis of *PARP1* (poly[ADP-ribose] polymerase 1) in vascular smooth muscle cells (VSMCs) seeded on control, early calcified, and late calcified ECM (extracellular matrix), normalized to VSMCs seeded on plastic, indicated by dashed line (n=5). **B**, Expression of osteogenic markers *Runx2* (Runt-related transcription factor 2), *BSP* (bone sialoprotein), *ALP* (alkaline phosphatase), and *BMP2* (bone morphogenetic protein 2) in VSMCs seeded on late calcified ECM and treated with a PARP inhibitor, PJ34, and an antioxidant, n-acetylcysteine (NAC), normalized to plastic control (n=6). **C**, Vitality of VSMCs seeded on late calcified ECM with PJ34 treatment was measured by reduced thiol levels and quantified as percentage healthy or unhealthy cells (n=9). **D**, Secretion of extracellular vesicles (EVs) from cells seeded on control or late calcified ECM was quantified relative to plastic control (dashed line) with or without NAC, wortmannin, and PJ34 treatment (n=8). **E**, Quantification of calcium content in VSMCs seeded on plastic, young ECM and senescent ECM (n=6). **F**, RT-qPCR analysis of *Runx2* and *ALP* in VSMCs seeded on young and senescent matrices, normalized to VSMCs seeded on plastic, indicated by dashed line (n=4). All data are presented as mean±SD. All experiments were performed in the 04-35F-11A donor.

Both oxidative stress and DNA damage signaling are key drivers of VSMC osteogenic differentiation.^30–33^ To understand the contribution of DNA damage response signaling to osteogenic processes in response to HA uptake, RT-PCR for *PARP1* (poly[ADP-ribose] polymerase 1) was performed and showed increased expression on the late calcified ECM compared with control and early calcified ECM (Figure 6A). VSMCs treated with either the ROS inhibitor, NAC, or PJ34, a PARP inhibitor, showed attenuated *Runx2* and *ALP* expression on the calcified ECM (Figure 6B). These drugs also improved cell vitality on the late calcified ECM (Figure 6C), indicating oxidative stress–induced activation of the DNA damage response pathway was increasing osteogenic gene expression. There was also a decrease in EV secretion from cells on calcified ECM when treated with NAC, wortmannin, or PJ34 (Figure 6D), indicating HA uptake–induced oxidative stress pathways and DNA damage signaling also drive increased EV secretion.^34^

We previously showed that healthy cells seeded onto ECM derived from senescent VSMCs acquired DNA damage.^25^ Comparison of the matrices showed that the senescent ECM is stiffer than a young control ECM and has increased COL10A1 (collagen type X alpha 1 chain) and EV-related protein deposition, similar to the calcified ECM. Therefore, we wondered whether senescent ECM could also induce an osteogenic response in the absence of mineral.^25^ Remarkably, we observed increased expression of *Runx2* and *ALP* on the senescent ECM. In response to Ca and P, VSMCs on the senescent ECM showed accelerated calcification compared with cells on plastic or young ECM; however, there was no further increase in osteogenic gene expression under these calcifying conditions (Figure 6E). Thus, the aged ECM niche is also able to directly promote robust osteogenic change in VSMCs in the absence of additional stimuli (Figure 6F).

### ECM Glycation Enhanced VSMC Calcification Through RAGE-Mediated ALP Activity

**Figure 7.**
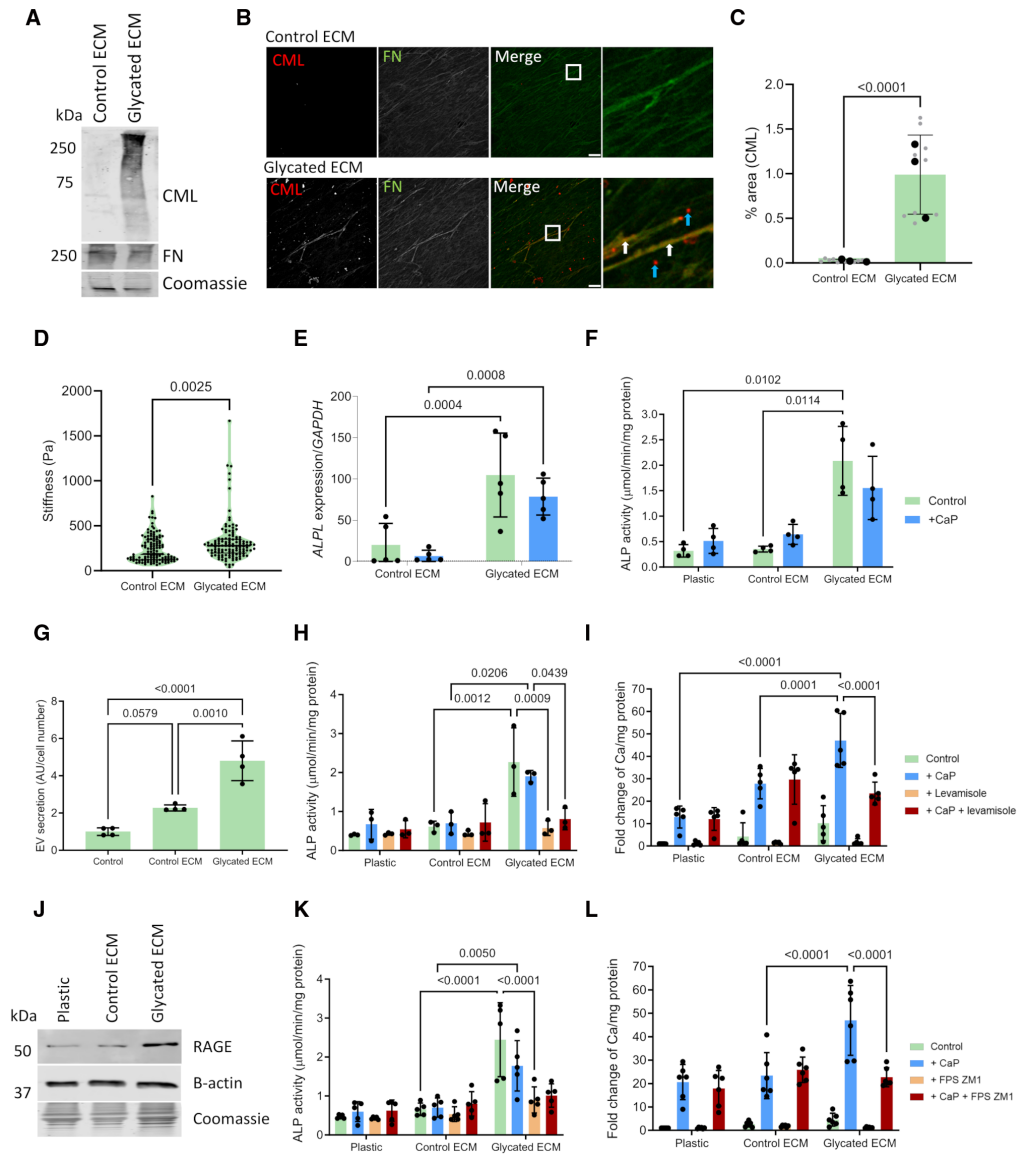
**ECM (extracellular matrix) glycation accelerates calcification through RAGE (receptor for advanced glycation end products) signaling. A**, Decellularized ECM was glycated by D-ribose treatment. Glycation was confirmed by Western blotting of a marker for advanced glycation end products (AGEs), N(6)-carboxymethyllysine (CML), in the ECM. Fibronectin (FN) and Coomassie were used as loading controls. **B**, Immunofluorescent (IF) staining for CML showed the accumulation of AGEs as fibril like, which colocalized with FN (white arrows) and as small, round deposits (blue arrows). Scale bar is 20 µm. **C**, Quantification of CML was performed to confirm the accumulation of AGEs in the glycated ECM. **D**, Atomic force microscopy was performed to measure stiffness of the control (n=121) and glycated ECM (n=126) from 03-38F-9A and 05-33F-5A donors. Gene expression (**E**; n=5) and enzymatic activity (**F**; n=4) of ALP (alkaline phosphatase) was quantified by reverse transcription-quantitative PCR (RT-qPCR) and colorimetric assay, respectively, in VSMCs seeded on the ECM under control or calcifying conditions (+CaP). **G**, Secretion of extracellular vesicles (EVs) was quantified from VSMCs seeded on control or glycated ECM under control conditions (n=4). **H**, Treatment with an ALP inhibitor, levamisole, confirmed a decrease in ALP activity in VSMCs seeded on the glycated ECM, under control and calcifying conditions (n=3). **I**, Calcium content was assessed by o-cresolphthalein assay in cells seeded on the matrices with levamisole treatment (n=5). **J**, Western blotting was performed to determine changes in expression of RAGE when VSMCs were plated onto plastic, control ECM, or glycated ECM (n=5). **K**, A RAGE inhibitor, FPS ZM1, was added for an ALP activity assay, under control or calcifying conditions (n=5). **L**, A calcification assay was performed to measure calcium content in VSMCs seeded on the ECM with FPS ZM1 treatment (n=6). All data are presented as mean±SD. All experiments were performed in the 04-35F-11A donor.

We next investigated whether glycation of the ECM could impact directly on VSMC osteogenic change. Decellularized ECM from healthy VSMCs was glycated using ribose, and glycation was confirmed by Western blotting and IF staining for the AGE marker N(6)-CML. Increased CML was detected as a smear by Western blotting, indicating modification of multiple matrisomal proteins (Figure 7A).^35^ On the matrix, CML staining colocalized, in part, with fibronectin, as well as forming small, round aggregates (Figure 7B). Quantification showed a significant increase in percentage area of CML in the glycated ECM compared with control (Figure 7C). AFM revealed that the glycated matrix was significantly stiffer than control ECM (Figure 7D).

Next, VSMCs were seeded onto plastic, control, or glycated ECM and treated with or without calcifying media. VSMCs plated on the glycated matrix did not show any reduction in cell viability (Figure S4A). RT-qPCR and ALP activity assay showed that the glycated matrix, independent of calcifying conditions, increased *ALP* expression and enzymatic activity in VSMCs (Figure 7E and 7F). However, the effect of glycation was exclusive to *ALP* as expression of other osteogenic markers such as *Runx2* and its downstream targets remained unchanged (Figure S4B). VSMCs seeded onto the glycated matrix also showed significantly increased EV secretion (Figure 7G). In response to calcifying conditions, VSMCs on the glycated ECM showed increased calcification compared with control ECM (Figure S4C).

To determine the contribution of ALP to glycation-induced calcification, VSMCs were treated with the ALP inhibitor levamisole. Levamisole attenuated ALP activity on the glycated ECM (Figure 7H) and caused a significant decrease in calcification of VSMCs only on the glycated ECM (Figure 7I). To determine whether increased ALP activity was via AGE signaling, Western blotting for RAGE was performed. This revealed a significant upregulation of RAGE expression in VSMCs seeded on the glycated ECM compared with plastic or the control ECM where levels were low (Figure 7J; Figure S4D). The addition of FPS ZM1, a RAGE inhibitor, to VSMCs on the glycated matrix led to a significant reduction in ALP activity (Figure 7K), which was associated with decreased calcification (Figure 7L). VSMCs seeded on the calcified ECM also showed increased ALP activity; however, RAGE expression in this context was not elevated (Figure S4E and S4F), suggesting ECM calcification and glycation induce ALP expression via distinct pathways.

## Discussion

In this study, we show that modifications to the ECM, common in aging and metabolic disease, directly drive pathological osteogenic change in VSMCs in the absence of other external stimuli. Exposure of VSMCs to mineral stress promoted compositional changes to the ECM and deposition of HA via EVs during the earliest phase of mineralization, while chronic exposure led to further changes in ECM composition, matrix stiffening, and the deposition of larger crystal aggregates. VSMCs seeded onto the calcified ECM underwent Runx2-mediated osteogenic changes, primarily driven by HA uptake and DNA damage signaling, and this same pathway was also activated in response to the senescent ECM.^25^ In striking contrast, nonenzymatic glycation of the ECM accelerated calcification via the specific induction of ALP expression and activity through AGE-RAGE signaling, demonstrating that selective osteogenic processes can be triggered in VSMCs by the ECM and that these can occur in the absence of mineral. These studies highlight a key role for metabolically induced ECM modifications including AGEs and mineral stress in VSMC calcification and demonstrate the importance of the ECM niche in accelerating osteogenic change.

### EVs as a Key Nidus for Calcification in Response to Mineral Stress

Electron micrographs of calcified human vascular tissue from aged patients and those with CKD have shown that calcification is first detectable in rounded EV structures.^15,36,37^ Consistent with these observations, we showed that exposure of VSMCs to mineral stress induced EV-mediated calcification and concomitant changes to ECM composition and properties, including increased collagen deposition and stiffness, confirming the relevance of this model to in vivo findings. We noted stepwise changes during the time course of calcification. Treatment of VSMCs with increased levels of Ca and P initially caused increased EV secretion, which preceded any changes in osteogenic gene expression, which occurred at a later stage when HA was already present. In the deposited ECM, HA crystals colocalized with the EV marker CD63 in the early stage, while at later stages, HA crystals formed around cores of aggregated CD63-positive EVs. Treatment with an EV secretion inhibitor decreased the amount of CD63 and HA deposited, substantiating the role of EVs in nucleating mineral.^15,38–40^ We also observed substantial retention of Ca in the minimally mineralized early ECM, which is consistent with in vivo studies in children with CKD where Ca accumulation in the vessel wall precedes the development of overt calcification and where EVs are the main nidus for mineralization.^37^

Mineral stress also induced changes in the composition of the ECM. At the early stage, there was increased deposition of several collagens, including COL1A1, COL3A1, and the cartilage-enriched COL10A1, as well as ECM-modifying enzymes and EV-associated proteins, consistent with entrapment of EVs within the modified ECM. Increased collagen and EV deposition persisted into the late stage of calcification with evidence for increased cell stress and death as calcification progressed. Increased abundance of type I and type III collagens and deposition of a collagen-rich matrix are thought to be hallmarks of vascular calcification.^41,42^ ECM compositional changes that enhance EV entrapment and aggregation are also key early events in the calcification process. Previous studies in cell-free systems have shown that collagen 1 and annexin A1 can act to aggregate EVs to enhance their calcification potential, resulting in the accumulation of micrometer-sized spherical calcifications.^18,43^ Taken together, these observations highlight the importance of EVs as a nidus for mineralization and clearly demonstrate an important role for early changes in ECM composition in promoting EV release, aggregation, and downstream pathways to calcification.

### Metabolic Stress and Aging Promote ECM Modifications That Directly Induce Osteogenic Differentiation and Accelerate Mineralization

Reseeding healthy VSMCs onto modified ECM rapidly induced phenotype changes that promoted mineralization. On the late calcified ECM, HA uptake led to the induction of oxidative stress and downstream DNA damage signaling, resulting in increased osteogenic gene expression. A previous study showed that addition of HA nanoparticles to VSMCs led to their internalization and trafficking into lysosomes, resulting in Ca release, dysregulation of Ca homeostasis, mitochondrial dysfunction, and ROS production.^44^ In this study, we showed that HA can be taken up directly from the ECM and that depletion of HA from the ECM with EDTA, or inhibition of its uptake via wortmannin, reduced osteogenic gene expression and enhanced cell vitality. Treatment with the antioxidant NAC or with the PARP1 inhibitor PJ34 also induced these changes. Consistent with the activation of Runx2 and its downstream targets in response to the calcified ECM, both oxidative stress and DNA damage signaling have been shown to increase Runx2 transcriptional activity and protein stability to promote osteogenic differentiation.^45^ This model provides the first evidence that the calcified ECM niche alone can directly initiate these osteogenic pathways and is consistent with numerous studies showing colocalization of DNA damage and mineral in vivo.^13,30,46^

We also observed upregulation of *Runx2* osteogenic gene expression when VSMCs were seeded onto the senescent ECM. Previous studies have shown that the senescent ECM can induce DNA damage and accelerate senescence in healthy VSMCs.^25^ This study suggests that the senescent ECM niche can also directly activate osteogenic pathways. The senescent ECM shows changes in composition, organization, and biophysical properties with some of the compositional changes, including increased deposition of COL10A1, collagen modifier P4HA2 (prolyl 4-hydroxylase subunit-α2), annexins (ANXA1, ANXA2, ANXA5, ANXA7, ANXA11), and the EV marker EEA1 (early endosome antigen 1), similar to those induced in response to mineral stress.^24,25^ Further work is now required to determine how these ECM changes promote DNA damage and phenotypic change.

In contrast to the activation of Runx2 on calcified and senescent ECM, seeding VSMCs on glycated ECM induced calcification specifically through RAGE activation and increased ALP activity. Treatment with inhibitors of RAGE or ALP attenuated osteogenic differentiation and calcification. RAGE expression was not increased in VSMCs seeded on the senescent or calcified matrices, suggesting this is a specific response to matrix glycation. Accumulation of AGEs is a characteristic of the diabetic and aging ECM, and RAGE has been shown to be upregulated in calcified and atherosclerotic plaques of patients with diabetes.^47,48^ Fibrillar collagens, laminin and fibronectin, are highly susceptible to glycation and cross-linking as the process of AGE formation affects proteins with long half-lives and with exposed lysine or arginine residues.^49^ Using nonenzymatic glycation of endogenously produced ECM, we could detect extracellular accumulation of AGEs and robust stimulation of RAGE expression in VSMCs with RAGE inhibition decreasing calcification and ALP activity. Binding of AGEs to RAGE activates signaling pathways via integrins, including MAPKs (mitogen-activated protein kinases), and previous studies have identified p38 MAPK as a mediator of AGE-induced calcification and highlighted the importance of the AGE–RAGE–p38 MAPK–ALP cascade in calcification of rat VSMCs treated with CML.^47,50,51^ RAGE signaling has been found to play a role in vascular calcification in a number of disease contexts.^52^ Interestingly, in this study, we found that RAGE was only increased in response to the glycated ECM and not in response to the calcified ECM, despite both activating ALP. In vivo, it is likely that VSMCs will be exposed simultaneously to >1 ECM modification; therefore, in the future, it will be important to incorporate multiple modifications to the ECM to determine whether they act synergistically to promote osteogenic change. It will also be essential to examine the downstream signaling activated by the glycated ECM and compare it with studies that have used other methods to activate RAGE and to cross-compare the signaling responses induced by the different ECM microenvironments.

### Early ECM Changes Create a Procalcific Microenvironment That Stimulates EV Release

In addition to the dramatic induction of heterogenous osteogenic pathways on the calcified, aged, and glycated ECM, we also observed a 4-fold increase in calcification potential in VSMCs seeded on the early, minimally calcified ECM. ECM compositional changes and modifications, including decreased elastin content, increased collagen deposition, and increases in enzymatic pathways such as MMPs and LOXs that promote matrix breakdown and cross-linking, were evident at this early stage. Although we did not observe any direct induction of osteogenic differentiation of VSMCs seeded on the early or HA-depleted ECM, we did observe increased EV release that may account for the increased ability of cells on these matrices to calcify. It is also well documented that ECM changes can potently impact on osteogenic change. COL1A1, COL2A1, fibronectin, as well as elastin and its fragments, can display osteopromotive effects and enhance osteogenic differentiation of vascular cells cultured on purified collagen or fibronectin in vitro.^53,54^ Thus, these ECM changes may also have contributed to the increased calcification observed.

Modified matrices were also stiffer than the ECM produced by control VSMCs. Removal of HA from the late calcified ECM significantly decreased its stiffness; however, it remained stiffer than the control ECM. Although the effects of matrix stiffness on VSMCs particularly during vascular calcification are not fully understood, VSMCs can undergo phenotypic switching and can alter gene expression in response to changes in matrix stiffness.^25,55,56^ However, in the context of this model, the changes in stiffness were not sufficient to directly induce VSMC osteogenic differentiation; however, in further studies, it will be feasible to introduce specific compositional and biophysical changes to the ECM to test this further.

### ECM as a Key Inducer of VSMC Osteogenic Processes

Controversies have remained concerning the factors that promote the initial nidus for mineralization in the vasculature and whether osteogenic differentiation of VSMCs is an early and essential component of the mineralization process or a later event activated in response to the calcified environment.^57–59^ Importantly, this study has highlighted that both mechanisms are likely to be occurring simultaneously in different disease contexts. We showed that EV-mediated calcification predominated when VSMCs were subjected to mineral stress. However, it is well documented that ECM components, such as elastin and collagen, can also directly mineralize via physicochemical processes that may be triggered by increased extracellular Ca released locally during cell necrosis. This was demonstrated in a recent study showing that poly(ADP-ribose), produced by PARP enzymes as a byproduct of the DNA damage response, accumulates in the ECM and can concentrate Ca ions to directly induce mineralization on collagen and elastin.^13^ Data from this ECM model suggest that once a mineral nidus is formed, whether on EVs or on ECM components, it can promote cell-mediated osteogenic processes via uptake of the mineral in a feed-forward cycle to accelerate calcification. In addition to this response to mineral uptake osteogenic process, we also demonstrated that ECM modifications alone can promote osteogenic differentiation of VSMCs with these pathways also accelerating calcification.

### Summary and Limitations

Current in vitro models rely by necessity on agents being added to cell culture in solution. Here, we describe a versatile 3-dimensional model that enables the study of key VSMC osteogenic processes in response to the ECM microenvironment and without the need for additional stimuli. The model can be adapted and specifically modified to test how the composition, individual components, and modifications of the ECM, including physical stress, stiffness, and breakdown, impact on cell phenotype and calcification enabling further dissection of the key outstanding questions in the field of vascular calcification. While some of the processes we have demonstrated in this model have been verified in vivo including activation of osteogenic signaling in VSMCs via the DNA damage response and by AGEs, there are still a number of outstanding questions that require further experimentation. Key is the dissection of the precise signaling pathways that promote VSMC osteogenic change in response to the nonmineralized ECM with these likely to involve complex mechanosignaling mechanisms.

## Article Information

### Acknowledgments

M. Whitehead and C.M. Shanahan contributed to conception; M. Whitehead, M. Faleeva, S. Cox, R. Oexner, L. Schmidt, and M. Mayr to acquisition of data; and M. Whitehead and M. Faleeva to analysis and interpretation of data. M. Whitehead and C.M. Shanahan wrote and revised the manuscript, and all authors provided final approval of the submitted version. The graphic abstract was made with Biorender.com. Microscopy was performed at the Wohl Cellular Imaging Centre and King’s College London.

### Sources of Funding

This study was supported by the British Heart Foundation Programme grant (RG/F/21/110064) to C.M. Shanahan and the KCL BHF PhD Programme grant (FS/15/66/32037).

### Disclosures

None.

### Supplemental Material

Figures S1–S4

Unedited Western Blots

Major Resources Table
